# Increased Thermal Conductivity in Metal-Organic Heat Carrier Nanofluids

**DOI:** 10.1038/srep27805

**Published:** 2016-06-15

**Authors:** Manjula I. Nandasiri, Jian Liu, B. Peter McGrail, Jeromy Jenks, Herbert T. Schaef, Vaithiyalingam Shutthanandan, Zimin Nie, Paul F. Martin, Satish K. Nune

**Affiliations:** 1 Environmental Molecular Sciences Laboratory, Pacific Northwest National Laboratory, Richland, Washington 99352, United States; 2 Energy and Environment Directorate, Pacific Northwest National Laboratory, Richland, Washington 99352, United States; 3 Fundamental Chemical Sciences Directorate, Pacific Northwest National Laboratory, Richland, Washington 99352, United States

## Abstract

Metal-organic heat carriers (MOHCs) are recently developed nanofluids containing metal-organic framework (MOF) nanoparticles dispersed in various base fluids including refrigerants (R245Fa) and methanol. Here, we report the synthesis and characterization of MOHCs containing nanoMIL-101(Cr) and graphene oxide (GO) in an effort to improve the thermo-physical properties of various base fluids. MOHC/GO nanocomposites showed enhanced surface area, porosity, and nitrogen adsorption compared with the intrinsic nanoMIL-101(Cr) and the properties depended on the amount of GO added. MIL-101(Cr)/GO in methanol exhibited a significant increase in the thermal conductivity (by approximately 50%) relative to that of the intrinsic nanoMIL-101(Cr) in methanol. The thermal conductivity of the base fluid (methanol) was increased by about 20%. The increase in the thermal conductivity of nanoMIL-101(Cr) MOHCs due to GO functionalization is explained using a classical Maxwell model.

Metal-organic heat carriers (MOHCs) are a new class of nanofluids developed by our group to enhance the thermodynamic and physical properties of a base fluid in which the metal-organic frameworks (MOFs) nanoparticles are uniformly dispersed^1^. MOFs possess unique properties including tunable porosity, extremely high surface area, and diverse metal/ligand combinations, which make them especially attractive^2^,^3^. MOF activity/properties can be further improved by grafting active materials^4^,^5^,^6^,^7^, pelletization^8^,^9^, and by post-synthesis modifications^10^,^11^. However, MOFs have longer linkers than any other inorganic class of porous materials (eg., zeolites). For an example, the number density of MOF-5 (2.46 × 10^28^ atoms/m^3^) is much lower than that of Sodalite (5.13 × 10^28^ atoms/m^3^) and Zeolite-A (4.10 × 10^28^ atoms/m^3^), and correlates directly into a lower volumetric specific heat^12^. The thermal conductivity in MOF can be positively related with the volumetric specific heat and to increase the thermal conductivity, producing MOHC composites is one of the simple, inexpensive and more efficient approaches^1^. Specifically, water and methanol have been identified as ideal base fluids for MOHCs due to their inherent stability as predicted by molecular dynamics simulations^13^,^14^. Nanoparticles of Prussian blue analogue Ni_3_[Co(CN)_6_]_2_ exhibited accelerated methanol adsorption kinetics^15^. MOHCs were also synthesized using well-dispersed nanoMIL-101 particles in water^13^. These stable aqueous nanofluids showed homogeneous distribution of nearly spherical MIL-101 nanoparticles. As emphasized in these studies on MOHCs, the long-term stability and increase in thermal conductivity are very critical for heat transfer applications.

While it is clear that an increase in thermal conductivity of a nanofluid will increase heat transfer through the fluid and therefore improves the heat transfer coefficients^16^,^17^,^18^, it is difficult to directly relate an improvement in thermal conductivity of a nanofluid to an increase in specific heat^19^. An increase in the specific heat of a nanofluid is advantageous in thermal storage and heat transfer applications, because it increases the amount of energy stored or absorbed per unit mass. While the focus of this paper is on increasing the thermal conductivity of MOHCs, an increase of nanofluid heat capacity is possible if the nanoparticles have a higher specific heat than the base fluid^20^.


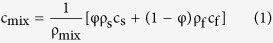


where c is the specific heat, for the mixture (mix), solid (s) and fluid (f), 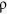 is the density and 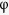 is the mass loading of nanoparticles in the fluid. Increasing the specific heat will result in an increase in the enthalpy gain of the nanofluid, and ultimately an additional increase in the work output (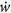)^1^.


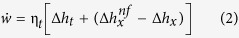


where η_*t*_ is the turbine efficiency, Δ*h*_*t*_ is the enthalpy change in the turbine, 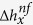
is the enthalpy change of the nanofluid in the heat exchanger and Δ*h*_*x*_ is the enthalpy change of the working fluid in the heat exchanger.

It should be noted that due to the small volumetric fraction of MOF nanoparticles considered in the current study, enhancement or degradation in the specific heat of the nanofluid is expected to be minuscule. Most importantly, increasing the thermal conductivity results in more rapid phase change and extraction or addition of heat via desorption or adsorption of the working fluid into or out of the nanoparticles.

On the other hand, MOF composites with different functional groups are of a greater interest due to their improved properties^7^. Specifically, MOFs functionalized with graphene oxide (GO) have attracted more attention because of their enhanced adsorption influenced by dense arrays of atoms and oxygen functionalities in GO^7^,^21^. MOF/GO composites combine the highly porous MOFs with atomically dense GO sheets to enhance the dispersive interaction in the open framework, which in turn increases the reactive adsorption of adsorbates including small molecules such as NH_3_, NO_2_, H_2_S, and CO_2_^7^,^21^,^22^. Moreover, graphene-based analogues can also increase the electrical and thermal conductivities of MOFs^22^,^23^. Specifically, MOF-5/graphite composites have shown a significant enhancement in the thermal conductivity up to 0.56 W.m^−1^.K^−1^ compared with that of the intrinsic MOF-5 (0.10 W.m^−1^.K^−1^)^23^,^24^. Due to these improved properties, MOF/GO composites have been synthesized by various methods and used for a wide range of applications including gas adsorption^21^,^25^,^26^,^27^,^28^,^29^, water adsorption^30^, CO_2_/CH_4_ separation^31^, catalysis^32^,^33^,^34^,^35^, electrochemical sensing^36^, liquid-phase adsorption^37^,^38^, and energy storage^39^,^40^. In our previous studies, stable MOHCs with neat MIL-101(Cr) nanoparticles have been easily synthesized using water by a simple hydrothermal method^13^. Hence, here we focused our attention on successfully functionalizing it with GO to improve the porosity, surface area, and thermo-physical properties.

## Results and Discussion

**Figure 1:**
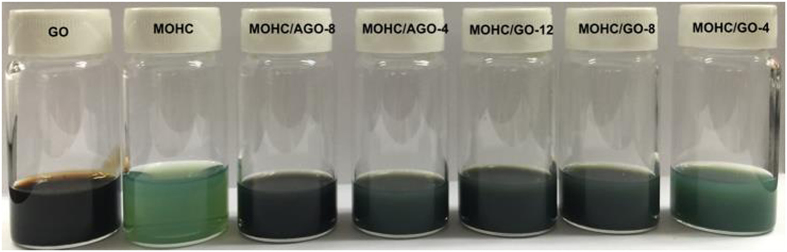
GO, MOHC, MOHC/AGO, and MOHC/GO nanofluids in methanol.

**Figure 2:**
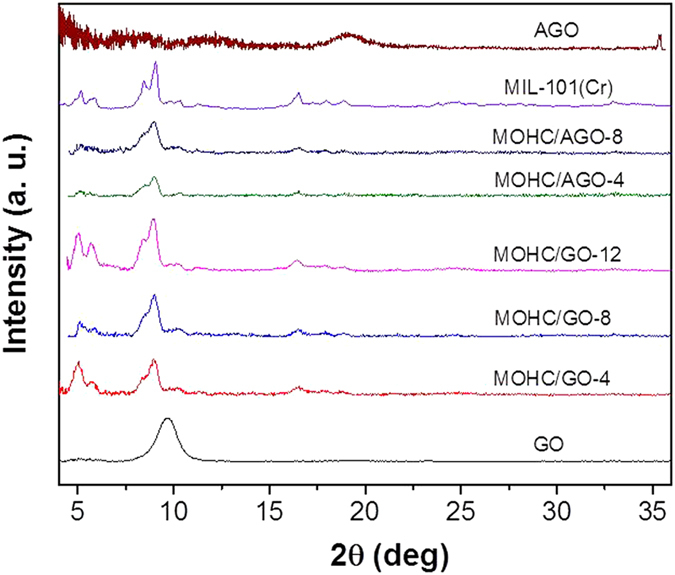
The powder X-ray diffraction (PXRD) patterns of MOHC/GO nanocomposites with different amounts of GO and amino GO. The PXRD patterns of intrinsic nanoMIL-101(Cr), GO, and AGO are also shown.

Various MOHCs containing a known amount of nanoMOF (MIL-101(Cr)), but with different amounts of GO (4, 8, and 12 mg) were synthesized. The stable suspensions of MOHC/GO composites in methanol are shown in Fig. 1. For nomenclature, the amount of GO in the composite is referred to such that MOHC/GO-4 indicates a MOHC composite containing MIL-101(Cr) and 4 mg of GO, which is about 0.0085 wt.%. We also prepared MOHC composites by functionalizing nanoMIL-101-(Cr) with GO containing amino groups. The amount of GO and amino GO (AGO) in each sample are listed in Table S1. Figure 2 shows the powder X-ray diffraction (PXRD) patterns of the MOHC/GO composites with different amounts of GO and AGO. The PXRD patterns of the intrinsic nanoMIL-101(Cr), GO, and AGO are also presented in Fig. 2 for comparison. The PXRD patterns of nanocomposites are dominated by reflections assigned to nanoMIL-101(Cr), indicating that the MOF crystallinity is preserved in the nanocomposites. These PXRD patterns are similar to the simulated XRD pattern of MIL-101(Cr)^41^. Some broadening of the peaks may be due to the nanocrystallinity of intrinsic nanoMIL-101(Cr) samples. Moreover, MOHC/GO nanocomposites with different GO contents lacked diagnostic peaks corresponding to pure GO, indicating that the GO was consumed during the formation of the MOHC-GO nanocomposites.

**Figure 3:**
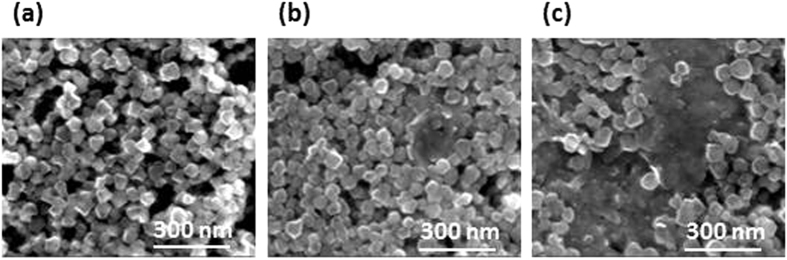
Scanning electron microscopy images of (**a**) MOHC/GO-4, (**b**) MOHC/GO-8, and (**c**) MOHC/AGO-4.

Figure 3 shows the high resolution scanning electron microscopy (SEM) images of MOHC/GO nanocomposites. SEM images of nanocomposites with different amounts of GO and AGO show the distribution of MIL-101(Cr) nanoparticles on the graphene oxide layers. The size of these nanoparticles with different shapes is in the range of 40–80 nm. No significant change in the size of MIL-101(Cr) nanoparticles in composites was observed with the increase in GO content compared to intrinsic nanoMIL-101(Cr) (Figure S1(a)). The high-resolution SEM images clearly show the different morphologies of nanoMIL-101(Cr) particles, which are nearly spherical or irregular in shape. The size and thickness of the pristine GO and AGO sheets are in the range of 1–5 μm and 200–300 nm, respectively as shown in Figure S1. X-ray photoelectron spectroscopy (XPS) was carried out on MOHC/GO composites to identify the elements and determine the elemental composition (Figure S2). XPS survey spectra indicated the presence of Cr, C, and O in all samples and some N in amino GO composites (Figure S2).

**Figure 4:**
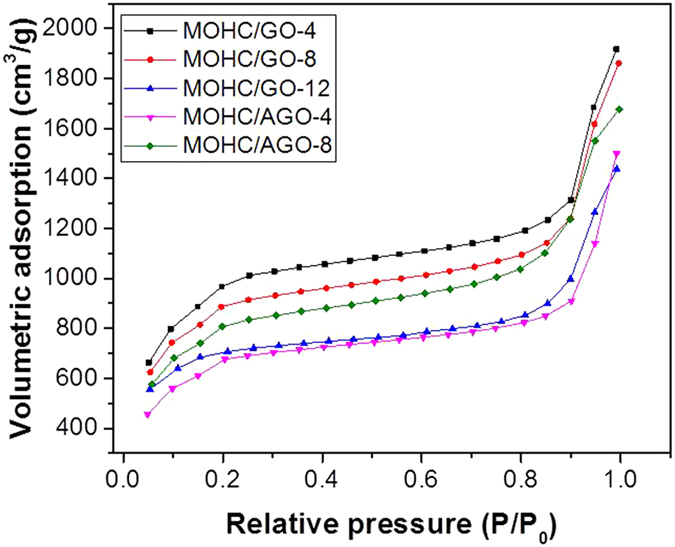
N_2_ adsorption isotherms of MOHC/GO nanocomposites.

Brunauer-Emmett-Teller (BET) surface area was determined using nitrogen adsorption-desorption measurements. Figure 4 shows N_2_ adsorption isotherms of MOHC/GO nanocomposites. These isotherms show typical type-I profiles based on the IUPAC classification with large uptakes of nitrogen at low relative pressure (P/P_0_ < 0.1), which is indicative of the presence of abundant micropores. Addition of 4 mg of GO during the synthesis of composite (MOHC/GO-4) resulted in the increased N_2_ adsorption compared to intrinsic MIL-101(Cr). The change in the N_2_ adsorption properties of composites compared to intrinsic nanoMIL-101(Cr) indicates the presence of GO/AGO in the composites. These isotherms revealed that the amounts of GO and AGO needed to obtain the optimum surface area for N_2_ adsorption are different. A steep increase in N_2_ adsorption at high relative pressures (P/P_0_ > 0.9) was observed with large hysteresis loops for MOHC/GO composites as shown in Figure S3, which is probably originated from nanophase or presence of meso/macro pores due to interstitial voids between particles. It is believed that some meso pores can be generated at the interfaces between GO layers and MIL-101(Cr) cages^30^. Thus, the amount of meso pores depends on the amount of GO/AGO in the composites, which results in some changes in the pore size distributions with an increase in GO/AGO content as shown in Figure S4. However, Figure S4 indicates similar pore structures in MOHC/GO composites regardless of the amount of GO/AGO. The nitrogen sorption isotherms of intrinsic nanoMIL-101(Cr) (Figure S5) show that it exhibits extremely high surface area of 2917 m^2^/g. Table 1 displays the BET/ Langmuir surface areas along with total/micro pore volumes of each nanocomposite. MOHC/GO-4 shows the largest surface area (BET–3160 m^2^/g) and highest pore volume (2.9 cm^3^/g). The surface area of MOHC/GO-4 is even higher than that of intrinsic nanoMIL-101(Cr) (2917 m^2^/g). Since GO does not exhibit a significant porosity^27^,^42^, the increase in the porosity of MOHC/GO-4 composite can be attributed to the generation of new pores at the interfaces between GO layers and MIL-101(Cr) cages^30^,^43^. Moreover, the delamination of GO layers and separation of MIL-101 cages are feasible, which can expose additional surface area in the composites^22^,^37^. The surface area and pore volume of the nanocomposites decrease with the increase in GO content from 0.0085 to 0.025 wt.%. In contrast, MOHC/AGO-8 shows higher surface area and pore volume than MOHC/AGO-4. This indicates that the optimum AGO content to obtain the highest surface area has not reached at the maximum tested value of 0.017 wt.%. As shown in the SEM images (Figure S1), the size of the GO and AGO sheets are significantly different. That means the volumetric density of AGO sheets is higher than that of GO sheets in the composites with a similar GO/AGO concentration, which leads to the generation of more pores at the interfaces between AGO layers and MIL-101 cages. Therefore it resulted in an increase in the surface area of MOHC/AGO composites with the increase in AGO content. The decrease in the surface area of MOHC/GO nanocomposites below a critical limit of GO content has also been observed for other MIL-101/GO and MOF-5/GO composites reported in the literature^22^,^30^,^37^,^44^. This decrease was attributed to the distortion of MIL-101(Cr) structure due to the electronic effect of surrounded GO^22^,^30^,^44^. The low contribution of GO to the surface area of composites at higher GO contents can also result in a decrease in total surface area of composites^37^. In our study, the optimum surface area was observed for the lowest amount of GO added in the MOHC/GO composites. For MOHC/AGO composites, the optimum surface area was observed for the highest AGO content added in the experiments. The optimum surface area in composites with different amount of GO is a result of the compromise between the surface area of nanoMIL-101(Cr), surface area of GO, and the generation of new pores at the interfaces between MIL-101 cages and GO sheets. All MOHCs exhibited behavior and capacities similar to those of intrinsic nanoMIL-101(Cr) during water adsorption at room temperature indicating that they had similar water adsorption and desorption mechanism. A rapid increase in the water adsorption capacity of MOHC/GO composites was observed from 40 to 60% of relative humidity (RH) (Figure S6–7). This steep increase can be attributed to the filling of both micro and meso pores with a similar hydrophilicity^45^. Thermogravimetric analysis (TGA) showed a significant decrease in the mass (~20–30 Wt%) upon heating indicating the loss of water adsorbed (Figure S8).

The thermal conductivity values of the MOHC/GO nanocomposites in methanol along with the reference samples including water, GO and AGO in water, methanol, and the intrinsic nanoMIL-101(Cr) in methanol are listed in Table 2. Since GO and AGO experienced aggregation in methanol and are not well dispersed, the thermal conductivities of GO and AGO in water (4 mg in 25 ml) are given as reference values. It is important to note that the focus of this discussion is to compare the thermal conductivity of composites with methanol and intrinsic nanoMIL-101(Cr) in methanol and not with GO or AGO. The thermal conductivity of the intrinsic nanoMIL-101(Cr) in methanol is 0.12 Wm^−1^K^−1^, which is lower than that of methanol due to the very low intrinsic thermal conductivity of MIL-101(Cr) (0.08 Wm^−1^K^−1^) as shown in Table S2. This value is in line with that reported for MOF-5 and its composite pellets (0.09–0.56 Wm^−1^K^−1^)^23^. However, there are a scant number of reports discussing the thermal conductivity of nanofluids containing MOFs. The MOHCs containing GO-4 in methanol showed a thermal conductivity of 0.19 Wm^−1^K^−1^, while the other nanocomposites containing AGO-4 show a value of 0.18 Wm^−1^K^−1^. Increasing the amount of colloidal GO in composites has little effect on thermal conductivity. These measurements confirm a ∼50% increase in thermal conductivity of MOHC/GO composites compared with intrinsic nanoMIL-101(Cr) in methanol. Moreover, the nanocomposites increased the thermal conductivity of methanol by ∼20%. These results are in line with thermal conductivity enhancements observed in nanofluids by over 33% and 32%, when 5% volume fraction of TiO_2_ nanoparticles and 7.5% volume fraction of CuO was added to water, respectively^46^,^47^. This significant enhancement in the thermal conductivity of MOHC/GO nanocomposites can be explained using the following theoretical model.

In general, increase in the thermal conductivity of nanofluids have been attributed to the convection caused by the Brownian motion of the nanoparticles in the base fluids, molecular level layering of the liquid in the liquid particle interface and effect of nanoparticle clustering^48^,^49^,^50^,^51^,^52^. However, the thermal conductivity of nanofluids depends on the type, volumetric fraction, spatial distribution, size, and shape of the nanoparticles as well as on the properties of the base fluid^48^,^49^. Various theoretical models have been proposed to predict the thermal conductivities of nanofluids^48^. The MIL-101(Cr) nanoparticles in nanocomposite MOHCs are nearly spherical and well dispersed within the graphene layers according to the SEM micrographs (Fig. 3). Therefore, a classical Maxwell model^48^ can be used to describe the relationship between the thermal conductivity of the nanofluid (*k*) and thermal conductivity of the base fluid (*k*_*f*_) as shown in the following equation.


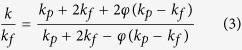


Here, *k*_*p*_ is the particle thermal conductivity and φ is the particle volumetric fraction. In our case, the base fluid is methanol and the mass loadings of the nanoparticles are well under 0.1%. Thus, we know that the particle volumetric fraction is far less than 1 and we can also assume that the thermal conductivities of MOHC/GO nanocomposites are much larger than that of methanol. Therefore the above relationship can be estimated as:


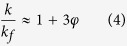


In the thermal conductivity measurements, the mass loading of MOHC/GO composites in methanol is about 0.02 wt.%. These experimental results show that the MOHC/GO nanocomposites have similar increased thermal conductivities regardless of particle mass loading. This may indicate that the nanoparticle volumetric fractions are very similar in all the tested nanofluids with MIL-101(Cr)/GO. In other words, the effective volumetric fraction reached a saturation point even in the most dilute case. Hence, an extra amount of GO/AGO in nanocomposites may do little to increase the thermal conductivity of the nanofluids as evident from the experimental results. In other words, even though the amounts of GO and AGO used to prepare the composites are different (Table S1), the real amount of GO and AGO mixed in the composites might be similar due to the dispersion limit of GO and AGO in the nanoMIL-101(Cr) suspension. Thus, a change in thermal conductivity of MOHC/GO composites was not observed with the increase in GO/AGO content.

Combining Equation (4) and experimental thermal conductivity values obtained for each sample, the effective volumetric fraction can be calculated as 0.078, which is much less than 1 as we assumed. It seems from the experimental results that the AGO functionalization did not affect the thermal conductivity of the nanofluids compared with MOHC/GO nanocomposites. Based on this theoretical analysis, MOHC/GO nanocomposites can increase the thermal conductivity of the base fluid (methanol) by ∼23% even at a mass loading of 0.02 wt.%. It further confirms the experimentally observed increase in thermal conductivity of methanol (∼20%), which is attributed to MOHC/GO nanocomposites. In contrast, further addition of composite nanoparticles above this mass loading may not further increase the heat transfer, which is attributed to the dispersion limit of the nanoparticles.

In summary, we report the successful synthesis of MOHCs containing nanoMIL-101(Cr)/GO composites that demonstrate the improved thermo-physical properties of base fluids without disturbing the crystallinity and porosity of nanoMIL-101(Cr). The PXRD patterns of the nanocomposites confirm the retention of MOF crystallinity. SEM micrographs showed 40–80 nm size MIL-101(Cr) nanoparticles, which are nearly spherical despite the presence of some particles with irregular shapes. An increase in the surface area and pore volume was observed for nanocomposites with less than 0.01 wt.% GO relative to the intrinsic nanoMIL-101(Cr). The surface area and pore volume of nanocomposites decreased with the increase in GO content from 0.01 to 0.025 wt.%. MOHCs in methanol with different amounts of GO/AGO showed a thermal conductivity of 0.18 Wm^−1^K^−1^. This is a 50% increase in thermal conductivity relative to the intrinsic nanoMIL-101(Cr) in methanol. According to the theoretical analysis of thermal conductivity based on the classical Maxwell model, MOHC/GO nanocomposites can improve the thermal conductivity of methanol by ∼23% even at a low mass loading of 0.02%, which also agrees with the experimental value of ∼20%. It was also suggested that a higher mass loading may not further improve the thermal properties due to the dispersion limit of MIL-101(Cr) nanoparticles.

## Methods

### Material synthesis

The MOHCs with nanoMIL-101(Cr)/GO and AGO were synthesized using the same procedure previously reported by our group^13^, except replacing the modulator by GO or AGO. Cr(NO_3_)_3_.9H_2_O (330 mg, 0.82 mmol), terephathalic acid (136.9 mg, 0.82 mmol), and varied amounts of GO and amino GO (4, 8, and 12 mg) were reacted in 25 ml of water at 180 °C for 4 hours in Teflon-lined autoclaves. The amount of GO/AGO in each sample and their names used in the discussion are listed in Table S1. After the reaction, the nanofluid was allowed to cool down to room temperature and centrifuged at a high speed to form a wet green pellet. Then 25 ml of DMF was added to the green pellet and the mixture was sonicated for 10 min at room temperature followed by heating at 135 °C for 24 hours in a Teflon-lined autoclave. The nanofluid was allowed to cool down to room temperature and centrifuged at a high speed. The resulted pellet was washed with methanol three times and the portions of the pellet were left in 25 ml of water, methanol, DMF (Figure S9), acetone, and THF to check the stability of nanofluids. Following the synthesis, all the MOHCs were characterized by PXRD, SEM, HIM, XPS, BET surface area measurements, VTI water-sorption analysis, TGA analysis, and thermal conductivity measurements.

### Powder XRD analysis

The powdered sample was characterized on a D8 Discover XRD unit equipped with a rotating Cu anode (0.154 nm), gobel mirror, 0.5 mm collimator, and 0.5 mm pin hole (Madison, WI). A GADDS area detector system positioned at a 2θ of 28.0° with a measured distance from the sample of 15 cm was used to capture diffraction images. Collection of individual XRD tracings required 200 seconds with power settings of 45 kV and 200 mA. Initially, images were processed with Bruker-AXS GADDS software before importing into JADE XRD software to obtain peak positions and intensities.

### SEM and HIM imaging

SEM imaging was carried out with FEI Helios 600 Nanolab instrument. Images were taken under immersion mode at a working distance of 4 mm and tilt angle of 52°.

### XPS analysis

X-ray photoelectron spectroscopy (XPS) was performed using a Kratos Axis Ultra DLD spectrometer, which consists of a high performance Al Kα monochromatic x-ray source (1486.6 eV) and a high resolution spherical mirror analyzer. X-ray source was operated at 150 W and the emitted photoelectrons were collected at the analyzer entrance slit normal to the sample surface. The data acquisition was carried out in hybrid mode with analysis area of 700 × 300 μm. The survey spectra were recorded at pass energy of 160 eV with 0.5 eV step size and high resolution spectra were recorded at pass energy of 20 eV with step size of 0.1 eV. The pass energy 20 eV in the 700 × 300 μm analysis area is referred to the FWHM of 0.59 eV for Ag 3d_5/2_. The charge neutralizer with low energy electrons was used to exclude the surface charging effects and the binding energy of C 1s at 284.8 eV was used as the charge reference. The chamber pressure was maintained at ~5 × 10^−9^ Torr during the measurements. XPS data was analyzed by CasaXPS software using Gaussian/Lorentzian (GL(30)) line shape and Shirley background correction.

### TGA analysis

Thermal stability of MOHC samples was investigated using a TG (NETZSCH TG 209 F1) under nitrogen (N_2_). About 5 mg of sample was heated under nitrogen; the gases evolved during heating were passed through a heated fused silica capillary into MS to obtain mass analysis during heating.

### BET surface area measurements

The surface areas of the sample powders were determined by the Brunauer–Emmett–Teller (BET) method and pore volumes were measured by Barrett–Joyner–Halenda (BJH) method, using nitrogen adsorption/desorption data collected with a Quantachrome Autosorb-6B gas sorption system on degassed samples. The fine powder samples were prepared for measurements by centrifuging the nanofluids, drying the pellets obtained after decanting the base fluid followed by grinding with a motor and pestle. The samples were degassed at 175 °C for 20 hours prior to the measurements. The nitrogen adsorption/desorption measurements were carried out at 77 K.

### Thermal conductivity measurements

The thermal conductivity measurements were conducted on a C-Therm thermoconductivity analyzer (Setaram Inc., Hillsborough, NJ) with a T306 sensor. Several droplets of nanofluids with different MOHC/GO composites were deposited on to the center of the sensor and a cap was attached on top of the sensor to minimize the evaporation. All the measurements were done at room temperature and the thermal conductivity values were obtained from five consecutive measurements.

### Water sorption measurements

The water vapor adsorption isotherms were obtained using a water vapor adsorption analyzer (VTI-SA+, TA Instrument, Florida, USA). Typically, a sample is regenerated at 150 °C withdry N_2_ flow for 12 h before the measurements. The relative humidity is achieved by controlling the ratio of the flow rates of the moisture stream out from the humidity generator using N_2_ as the carrier gas.

## Additional Information

**How to cite this article**: Nandasiri, M. I. *et al.* Increased Thermal Conductivity in Metal-Organic Heat Carrier Nanofluids. *Sci. Rep.* **6**, 27805; doi: 10.1038/srep27805 (2016).

## Supplementary Material

Supplementary Information

## Figures and Tables

**Table 1:** Surface area and pore volume measurements of different MOHC/GO samples.

Sample	Surface area (m^2^/g)		Pore volume (cm^3^/g)	
	BET	Langmuir	Total	Micropore
MOHC/GO-4	3160	5225	2.9	1.74
MOHC/GO-8	2823	4656	2.74	1.57
MOHC/GO-12	2109	3429	1.85	1.2
MOHC/AGO-4	2156	3545	2.27	1.19
MOHC/AGO-8	2576	4290	2.44	1.44

**Table 2:** Thermal conductivity values of MOHC/GO nanocomposites and the reference samples.

Sample	Thermal conductivity (Wm^−1^K^−1^)
Water	0.5
GO^#^	0.54
AGO^#^	0.55
Methanol	0.15
MIL-101(Cr)*	0.12
MOHC/GO-4*	0.19
MOHC/GO-8*	0.18
MOHC/GO-12*	0.18
MOHC/AGO-4*	0.19
MOHC/AGO-8*	0.18

^#^in water, ^*^in methanol.
