# Supplementary material for: Increased Thermal Conductivity in Metal-Organic Heat Carrier Nanofluids
Source: Sci Rep. 2016 Jun 15;6:27805. doi: 10.1038/srep27805 (PMC4908600; doi:10.1038/srep27805)
Supplement: Supplementary Information [file srep27805-s1.doc]

Supplementary Information

Increased Thermal Conductivity in Metal-Organic Heat Carrier Nanofluids

Manjula I. Nandasiri,^1^ Jian Liu,^2^ B. Peter McGrail, ^2^ Jeromy Jenks,^2^ Herbert T. Schaef,^3^ Vaithiyalingam Shutthanandan,^1^ Zimin Nie,^2^ Paul F. Martin, ^2^ and Satish K. Nune*^, 2^

^1^Environmental Molecular Sciences Laboratory, Pacific Northwest National Laboratory, Richland, Washington 99354, United States

^2^Energy and Environment Directorate, Pacific Northwest National Laboratory, Richland, Washington 99354, United States

^3^Fundamental Chemical Sciences Directorate, Pacific Northwest National Laboratory, Richland, Washington 99354, United States

*Corresponding author: satish.nune@pnnl.gov

Table of Contents

Table S1 S3

Table S2 S3

Figure S1 S4

XPS analysis S5

Nitrogen sorption measurements S6

Water sorption measurements S7

TGA analysis S9

Figure S9 S10

| Sample | Amount of GO/AGO (mg) | GO/AGO (wt.%) |
| --- | --- | --- |
| MOHC/GO-4 | 4 | 0.0085 |
| MOHC/GO-8 | 8 | 0.0168 |
| MOHC/GO-12 | 12 | 0.0250 |
| MOHC/AGO-4 | 4 | 0.0085 |
| MOHC/AGO-8 | 8 | 0.0168 |

Table S1. The amount of GO/amino GO in the synthesized MOHC/GO nanocomposites.

| Measurement # | Sensor | Effusivity (WsÂ½/mÂ²K) | k (W/mK) |
| --- | --- | --- | --- |
| 1 | T306 | 180.465 | 0.0806 |
| 2 | T306 | 180.972 | 0.0808 |
| 3 | T306 | 180.122 | 0.0805 |
| 4 | T306 | 182.079 | 0.0811 |
| 5 | T306 | 182.198 | 0.0812 |
| 6 | T306 | 180.139 | 0.0805 |
| 7 | T306 | 180.443 | 0.0806 |

Table S2. The intrinsic thermal conductivity of nano MIL-101-(Cr).


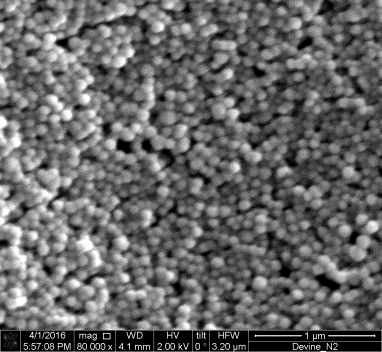


(a)


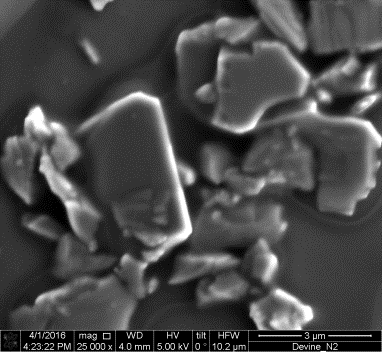


(b)


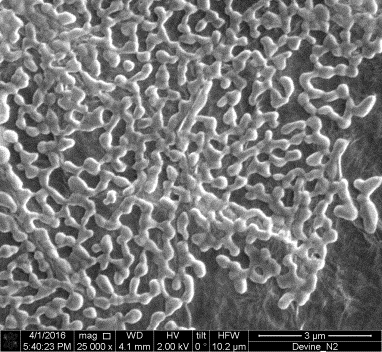


(c)

Figure S1. Scanning electron microscopy images of (a) intrinsic nanoMIL-101-(Cr), (b) GO, and (c) AGO.

**XPS analysis**

X-ray photoelectron spectroscopy (XPS) was carried out on MOHCs to identify the elements and determine the elemental composition. XPS survey spectra of MIL-101(Cr)/GO MOHCs are shown in Figure S2, indicating the presence of Cr, C, and O in all samples and some N in amino GO composites.

Figure S2. The XPS survey spectra of MOHCs with MIL-101(Cr)/GO nanocomposites.

**Nitrogen sorption measurements**

Figure S3. N_2_ adsorption- desorption isotherms of MOHC/GO-4 and MOHC/AGO-4 nanocomposites.

Figure S4. Pore size distributions of MOHC/GO nanocomposites.

Figure S5. N_2_ adsorption- desorption isotherms of intrinsic nanoMIL-101(Cr).

Water sorption measurements

Figure S6 shows the water adsorption isotherms of MOHC/GO nanocomposites at 298 K. All the MOHCs show a similar behavior for adsorption of water vapor indicating a similar water adsorption mechanism. These isotherms can be identified as type-V with three different regions in the water adsorption. At low relative humidity (RH < 40%), isotherms show a linear increase in the water adsorption capacity with the increasing RH, wherein the water molecules are bonded to hydrophilic centers. A rapid increase in the water adsorption capacity of MOHC/GO composites from 20 to 90 wt.% was observed with a further increase in RH from 40% to 60%. The presence of mesopores also leads to water adsorption in a higher relative humidity region. Beyond 60% of RH, water adsorption capacity still shows a slight linear increase with the increase in RH at a rate similar to that of low humidity region before reaching the optimum value. This slight increase in the high humidity region can be attributed to the adsorption of water molecules in the interparticulate voids of the powder. At ~80% of RH, ~100 wt.% of water was adsorbed by the nanocomposites. Water adsorption-desorption isotherms for MOHC/GO composites at 298 K are shown in Figure S7. When the water adsorption reaches a saturation point, desorption isotherm follows a different path from the adsorption isotherm. Thus, large hysteresis loops were observed in the range of 30-60% of RH for all the samples with desorption isotherm is above the adsorption isotherm for each sample as shown in Figure S5. These hysteresis loops imply the strong hydrogen bonds between water molecules, which led to slow water desorption.

Figure S6. The water adsorption isotherms for MOHC/GO nanocomposites.

Figure S7. The water adsorption and desorption isotherms for (a) MOHC/GO-4 (b) MOHC/GO-8 (c) MOHC/GO-12 showing hysteresis loops for each sample.

Thermogravimetric analysis (TGA)

Thermogravimetric analysis (TGA) was carried out on the MIL-101(Cr)/GO MOHCs in the temperature range of 303 to 673 K in nitrogen atmosphere at a rate of 4 K/min to study the change in mass due to the removal of water and different gases. TGA water plots of the nanocomposites are shown in Figure S8, which outlines the weight loss of the samples due to the removal of water during the annealing in nitrogen atmosphere. All the samples showed a significant decrease in the mass about 20-30 % due to the dehydration after heating up to 100°C. TGA plots further indicate the removal of most of the water in the samples after heating at ~60°C. The MIL-101(Cr)/GO-12 sample shows the smallest mass change (~ 20% at 100°C) compared to the other samples.


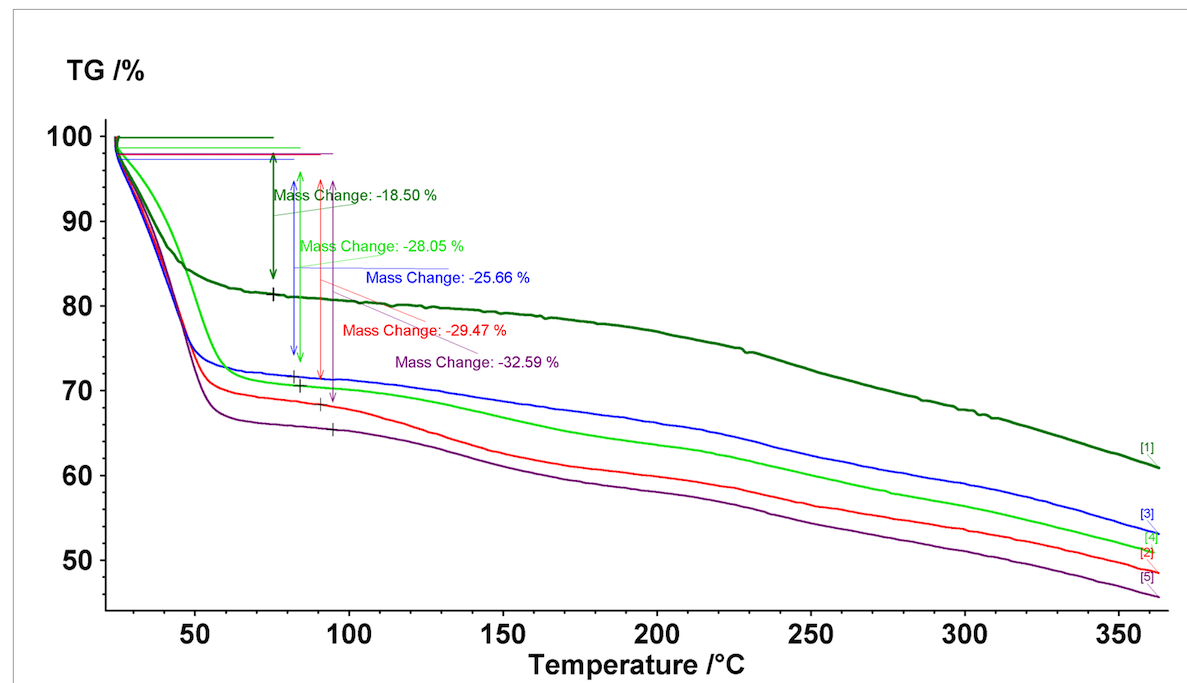


Figure S8. TGA (water) plots of (1) MOHC/GO-4 (2) MOHC/GO-8 (3) MOHC/GO-12 (4) MOHC/AGO-4, and (5) MOHC/AGO-8 nanocomposites indicating the weight loss due to the dehydration during the heating in nitrogen atmosphere.

Figure S9. (a) MOHC/GO-4 and (c) MOHC/AGO-4 stable nanofluids in DMF.
